# Chicago Medical Society, Meeting of Dec. 16

**Published:** 1879-02

**Authors:** Roswell Park


### Society Reports.

### ARTICLE VIII.

### CHICAGO MEDICAL SOCIETY.

Reported for the JOURNAL AND EXAMINER, by DR. ROSWELL PARK.

At a meeting held Dec. 16, 1878, Dr. John Bartlett read a paper on Placenta Prævia, suggesting an original method of treatment, a full abstract of which we present:

DR. BARTLETT introduced the subject matter of his paper, by a reference to the several plans for the management of placenta prævia commonly in vogue. He then proceeded to state the objections to these methods, quoting Lee and Simpson, as declaring the tampon an uncertain and treacherous aid, and adding: "It is quite possible, that, by damming up the blood, the tampon subjects the surface of the placenta to the hydraulic pressure of the mother's circulation with the result of separating it from the uterus too early, or to an unnecessary extent." In one case, Dr. Bartlett thought he had observed the prolonged use of the plug to lead to putrefaction of the retained blood and consequent septicæmia. It was a serious objection to the tampon that, when its removal for any reason became necessary, from the moment of its disturbance to the secure adjustment of the new plug, a greater or less loss of blood occurred.

In reference to the use of Barnes' dilators, for arresting bleeding, and dilating the os, he said: "They tend to expand in the direction of least resistance, and may thrust the placenta off from the cervix to a greater extent than may be necessary." In regard to the puncture of the membranes and the use of ergot, Dr. B. objected that the loss of the liquor amnii and the ergotic contraction of the uterus, in case necessity arose for changing the position of the child, render turning difficult and may cause a delay injurious or even fatal to mother or child.

Dr. Bartlett having quoted from the summary given by Dr. Barnes in his "Obstetric Operations," passages having a bearing on the matter of his paper, said : " I conceive that Dr. Barnes is correct as to there being a period or stage when the flooding is spontaneously arrested, at least to a very considerable extent," but his view as to the cause of this arrest differed greatly from that of Dr. Barnes, who says, " when the dilatation has reached the stage at which the head can pass, the intermitting active uterine contractions arrest the hæmorrhage." Dr. Bartlett referred the arrest of bleeding mainly to the altered relation between the uterine vessels and the uterine tissue, through which they pass, brought about by the change undergone in the cervix from the state of contraction to that of more or less dilatation, a change not to be described as one of " contraction," " retraction," or " dilatation," but rather as a kind of stretching. " Were we able," he said, " to pierce this thick rubber band with hollow tubes, here represented by threads extending from edge to edge, it is plain that while it is as now unexpanded, unstretched, the flow of a fluid through the tubes would be easy and unchecked, but were I then to draw upon the band in this manner (stretching it) so as largely to increase its diameter, it can readily be conceived that vessels passing through its substance would be compressed, and the passage of fluid through them greatly retarded or arrested. It is thus, I think, by that active change in the os uteri, so resembling stretching, termed dilatation, that the vessels within it are impinged upon, and bleeding checked."

Dr. Bartlett continued : " It would appear then that in placenta prævia, hæmorrhage is liable to occur until the cervix uteri is fully dilated ; that when that occurs, at least in actual practice, where the head or body of the child presses into, or is drawn into the cervix, bleeding ceases. That to await this dilatation, even when the tampon is used, is dangerous ; that dilatation is delayed by the attachment of the placenta to the cervix, acting in some degree as an unyielding splint upon it, and that the loosening of the splint, the artificial detachment of the placenta, is a step gained toward dilatation; that while this separation of the placenta over the cervix is desirable, it is important that it should occur to the degree necessary for the dilatation of the os only, inasmuch as the breathing space of the child is lessened, and the prospect of its being born alive is diminished in exact ratio to the extent of placental detachment."

As an improved method of effecting dilatation, Dr. Bartlett proposed the following plan:

"Perforate the presenting placenta by means of an instrument carried upon the last joint of the index finger, resembling a tailor's thimble, provided with a serrated margin resting upon and extending slightly beyond the finger-nail. Perforation having been effected, the opening made is closed by the point of a finger until by means of the left hand a Hobb's dilator, shaped like a truncated cone, with the base upwards, and having a diameter when expanded as great as that of the hand, is slipped into the perforation. Hobb's dilators consist of the usual arrangement of tubes and rubber bags with this additional device—they are covered with a sack of stout silk of any shape desired, with the effect of causing them, when once pinched into a strictured orifice to continue to distend it with gentle force, till the contraction shall be thoroughly dilated, and this, without danger of over-expansion of the parts of the sack above or below the stricture. They are, because of the thinness of their walls, capable of being introduced into narrow appertures, and they may be provided with an index by means of which the moment of their complete dilatation may be known. Such a dilator, being passed through the perforation in the placenta, is quickly expanded so as to bear upon the margin of the opening with the view of preventing the escape of liquor amnii and of arresting bleeding from the placental vessels. Then by gentle hydrostatic pressure, the os uteri, and with it the opening through the placenta, is dilated to a size sufficient to readily admit the hand, slight traction being made meanwhile on the staff of the dilator so as to secure the pressure of the placenta against the cervix with the view of limiting detatchment as far as practicable, and at the same time of arresting hæmorrhage from the fœtal and uterine vessels alike.

Dilatation being fully effected, as indicated by the register, the operator diminishes the tension of the dilator by lowering the fountain of water, so that the hand may be easily insinuated between the bag (its contents regurgitating under the pressure) and the apposed margins of the cervix and placenta.

The hand is introduced, the dilator being withdrawn, so soon as the arm is felt to fill the os, the feet brought down and delivery effected as promptly as practicable.

By this method, Dr. Bartlett believed, the principle of the tampon would be applied with the greatest certainty and efficiency—that advantage is gained by introducing the plug once for all, no removals or changes being required in the process of dilatation. He thought the tampon would act in such a manner that instead of tending to separate to an unnecessary and undesirable extent, the placenta from the cervix, it would, on the contrary retain parts of it in apposition and in vital connection, which would by other procedures be separated.

The shape of the tampon and its position relatively to the placenta and cervix would tend to crowd these structures together in such manner as to prevent to the greatest possible extent bleeding from either.

The peculiarity of the dilator in expanding to a size definite and known and in automatically indicating when complete expansion was reached, enabled the practitioner to proceed to introduce the hand without fear of using violence and to turn, the liquor amnii being preserved, with ease, and without undue apprehension lest a dangerous delay result from an insufficient opening in the placenta or an inadequate dilatation of the os.

It has been objected to this method that perforation of the placenta, early suggested, has been abandoned because it is difficult of execution, because the insufficient opening in the placenta is sometimes an obstruction to delivery, and finally, that it is dangerous to the child in consequence of the wounding of the placental and uterine vessels.

The first objections do not apply to this plan, and in regard to the last it may be said that though the perforation of the afterbirth is generally condemned by writers, there are yet quite an array of authors who have found reason to recommend it." Here the essayist recited a list of names, concluding with those of

Denman, Blundell, Bard and Bedford. It was quite possible that in placenta prævia centralis perforation of the after-birth would involve wounding the largest placental vessels. The writer had no disposition to ignore this objection, but from certain *a priori* physiological reasons, he was of the opinion that the implantation of the cord at the point in the placenta corresponding to the os uteri would prove to be a rare occurrence.

The Doctor concluded by citing a fact which he regarded as rather interesting, analogically, than actually bearing upon the plan of treatment proposed, namely, that in the ungulates, as the mare and the sow, which have what the comparative anatomists denominate a *diffuse* placenta, an enclosed sac of umbilical vessels entirely surrounding the young, perforation of the placenta is essential to birth.

DISCUSSION.

At the conclusion of the paper, discussion being invited, Prof. Fitch commended the idea as being ingenious and possibly the best plan yet devised. But he feared that separation would be rather favored. When bleeding had occurred there must be more or less separation, and what was to become of the ruptured vessels? Would not the danger of uterine hæmorrhage be increased?

DR. HARCOURT inquired if Dr. Bartlett proposed to limit the plan proposed to the management of placenta prævia centralis? In case of lateral presentation, could the method suggested be depended upon for arresting the bleeding of placental vessels torn in slitting the placental edge?

DR. PAOLI held that no positive rule could be laid down for general adoption. He preferred the tampon to an entirely new method. He had six cases, in three of which, by aid of the tampon, he had succeeded.

DR. SAWYER thought it difficult to know what instructions to give a class when doctors thus disagreed. He considered it better

NOTE.—Dr. Bartlett suggested to the reporter that an instrument for perforating the placenta could be extemporized from a large thimble, by partially detaching its top from the conical portion by filing around about two thirds of its circumference, and thrusting it outward so that it stand in a plane parallel to the axis of the thimble. This portion being filed smaller and serrated on the edge, the instrument was ready for use. Muscular tissue and even thin board can be readily cut through by repeated sawing movement of the index finger armed with this device. A large sized Barnes' Dilator or a pig's bladder could be substituted for Hobb's Dilator.

for younger practitioners and students to have some precise idea of what should be done in such cases. If they were to go on the general rule of "using their own judgment," he feared they would lose every case. In seven years' practice he had had three cases ; two of them complete or central, both of which he lost. His first case died of syncope from a rupture of the cervix and uterine tissue, as proved by autopsy. The site of placental adhesion was as rotten as wet paper. He thought the descent of the head, or, possibly, the introduction of the hand, thus ruptured it. His first case also had some laceration and died of puerperal septicæmia and mania. His method was to use the tampon. He felt sure that the hæmorrhage is largely from the placental as well as the uterine tissue; also that if he had perforated the centre of the presenting surface, he would have wounded the largest placental vessels, instead of avoiding them, as Dr. Bartlett thought might be the case. His autopsy had taught him that. He thought there was little comparison between the gravity of a partial and a central implantation. As a general instruction he would say "plug, and either turn or use the forceps."

The President, DR. INGALS, thought that the mere presence of clot partially or wholly filling the vagina might be made a sufficient tampon and thus serve a good purpose.

DR. BARTLETT replied : To the one or two members who would seem disposed to repudiate predetermined action in the management of placenta prævia, and even to discard " anything new," suggested for its treatment, he would simply cite the language of Dr. Simpson in his remarks prefatory to his paper " On the Spontaneous Expulsion and Artificial Extraction of the Placenta before the Child " : " Mothers who are the subjects of placental presentations are submitted to as great peril of life from this obstetric complication, as they would be if seized with yellow fever or the cholera. Looking at these results, it will, we believe, be readily conceded that any attempt to diminish this fearful maternal mortality in placenta prævia is entitled, at least, to the consideration of the obstetric profession." In reply to Dr. Fitch's question, he would repeat the opinion expressed in a portion of the paper read, he believed, when Dr. Fitch was out of the room ; that the pressure of the tampon from above downward, crowding together the apposed surfaces of the placenta and uterus, would prove the surest method of either preventing or arresting hæmorrhage. In answer to Dr. Harcourt, he stated that the method was intended for central, lateral, and so far as the use of the peculiar dilator was concerned, incomplete implantations of placenta prævia. A distinguished obstetrician, of this city, had, in conversation, put the same question as Dr. Harcourt had advanced; he believed that the tampon used as advised would be as likely to control bleeding under these circumstances as any other means, but a satisfactory answer to the Doctor's question, could be given only after trial. Dr. Sawyer's case, where he found the origin of the cord corresponding exactly with the os uteri would seem to contravene the theory advanced in the paper as to the improbability of such an occurrence. In reply to the question of the President, whether he had tried the plan suggested, Dr. Bartlett stated that he had not; that the method had occurred to him within a few months.

During the evening the following interesting facts were stated: Dr. C. T. Parkes' *first* labor case was one of placenta prævia; while Dr. Fitch had but *two* cases in twenty-seven years practice, Dr. Mary Thompson had seen two cases, on adjoining beds, in the same hospital ward within a single week.

